# “No visible signs of pregnancy, no sickness, no antenatal care”: Initiation of antenatal care in a rural district in Northern Ghana

**DOI:** 10.1186/s12889-019-7400-2

**Published:** 2019-08-13

**Authors:** Agnes Millicent Kotoh, Michael Boah

**Affiliations:** School of Public Health, University of Ghana, LG 13 Legon, Ghana

**Keywords:** Pregnancy, Antenatal care, First trimester, Second trimester, Traditional rites, Ghana

## Abstract

**Background:**

Attending antenatal care (ANC) early contribute to better birth outcomes. Studies have shown that many pregnant women in Sub-Saharan Africa do not initiate ANC early (i.e. in the first trimester). This study determined the gestational age of pregnancy at first ANC attendance. It also explored factors that influence initiation of ANC.

**Methods:**

This cross-sectional study, conducted in Ghana, used mixed methods to collect data from women aged 15–45 years who delivered 6 months prior to the study. Crosstabs, chi-square test and logistic regression were used to analyse quantitative data. Also, 33 participants were engaged in focus group discussions (FGDs). Thematic content analysis was used to develop themes from the data.

**Results:**

Of the 431 participants, 8.9, 8, 25.4, 45.3 and 10.7% started ANC in the first, second, third, fourth and fifth months of pregnancy respectively. Formal education, employment and number of living children were predictors of initiating ANC early; by 12 weeks of gestation. Women who attained primary, junior high, secondary education and above had 5.6, 57.5 and 163.2 higher odds respectively of initiating ANC in the first trimester compared to women with no education (*p* ≤ 0.05). Women with two, three and four to nine living children were 4.1, 3 and 3.5 times respectively more likely to access ANC early compared to primigravidae women. However, women with five or more children and primigravidae women are more likely to initiate ANC late; after 12 weeks gestation. The FGD data also show that most of the participants initiated ANC late. Two themes: visible signs of pregnancy and or sickness influence ANC attendance in the first trimester. The themes that explain late initiation of ANC are: healthy, do not value the benefits of early ANC attendance, desire to avoid embarrassment associated with the pregnancy, unplanned pregnancy, indirect cost of accessing ANC and traditional rites and practices.

**Conclusion:**

Contextual factors influence ANC initiation. Investment in female education, intensification of health promotion activities by health workers, non-governmental organisations, community and religious leaders to sensitise communities on the benefits of initiating ANC at the onset of pregnancy is needed to improve first trimester attendance.

**Electronic supplementary material:**

The online version of this article (10.1186/s12889-019-7400-2) contains supplementary material, which is available to authorized users.

## Background

Antenatal care (ANC) prevents and reduces pregnancy and delivery related complications such as postpartum haemorrhage, hypertension, pre-eclampsia, eclampsia, sepsis, spontaneous abortion and obstetric fistula which are the leading causes of maternal morbidity and mortality globally and in Ghana [1–3]. During ANC, pregnant women are screened and treated for sexually transmitted infections (STIs) including HIV, preeclampsia and anaemia. Drugs such as iron and folic acid supplements to treat and prevent anaemia, prophylaxis against malaria, tetanus toxoid, as well as education on how to improve maternal and foetal outcomes are also provided. The World Health Organisation (WHO) recommends ANC initiation during the first trimester of pregnancy (i.e. the first 12 weeks of gestation) and a minimum of four visits before delivery [4–6]. These initiatives have improved pregnancy and birth outcomes [1, 4–8] and can be described as the foundation of maternal and neonatal healthcare.

Several studies have shown that late initiation of ANC (i.e. after 12 weeks) is a significant risk factor for all maternal deaths and recommend pregnant women should have their first ANC appointment by 12 weeks in order for all screening to be completed and risk factors addressed before 21 weeks of gestation for best pregnancy outcomes [4–6, 9]. The United Kingdom Maternal Mortality Reports show that late initiation of ANC is a significant risk factor for all maternal deaths. They recommend that pregnant women should have their first ANC appointment 10–12 completed weeks of pregnancy in order for all screening to be completed and risk factors addressed before 21 weeks of gestation [9–11]. The Ghana Health Service (GHS) also recommends that the first ANC attendance should preferably be in the first trimester for timely identification of problems and management, giving appropriate advice to protect the mother’ health and foetus’ optimal development for best pregnancy outcomes [2].

However, evidence shows that many women especially in sub-Saharan Africa do not access ANC services in the first trimester of pregnancy with others attending only once [1, 12–21]. The GHS reports that 45.1% of pregnant women in the country started ANC in the first trimester in 2013 [2]. The Ghana Demographic and Health Survey (GDHS) also reports that the average gestational age of pregnancy of first ANC attendance was 3.6 months in 2014. An Ethiopian study also reports that nearly 42% of women started ANC in the first trimester of pregnancy in 2012 [21].

Many low and middle income countries (LMICs) are using social health insurance schemes (SHISs) to increase access to maternal health services. In Ghana, pregnant women enrol into the national health insurance scheme (NHIS) without paying premium and registration fees and have free access to the ANC and other services. The NHIS was introduced by government in 2004 to improve access to healthcare. It operates a nationalised system of service provision and financing without co-payment and requires an annual payment of registration fee of GHC 5.00 (about US1.00) and GHC30.00 (about US$7.00) as premium. Healthcare services including medicines are obtained from accredited public health, faith-based, quasi-government and some private health facilities, pharmacy and chemist shops [22] who operate under contract with the National Health Insurance Authority (NHIA). All persons including pregnant women who do not have valid NHIS cards pay out-of-pocket at the point of accessing health services including ANC.

With the free access to ANC and the expansion of health services especially community-based health planning and services (CHPS) compounds^1^ nationwide, it is expected that all pregnant women will initiate ANC early but this has not happened. The 2014 GDHS shows that 64% of women in Ghana initiate ANC by the fourth of pregnancy in 2014 with varied regional and district rates [15]. Facility data shows that 34.7, 39.4, 35, 31.7 and 37.5% of pregnant women in the Builsa South District initiate ANC in the first trimester in 2010, 2011, 2012, 2013 and 2014 respectively [16]. These statistics show that despite the removal of geographical and financial obstacles to ANC, there are significant barriers to first trimester ANC attendance in Ghana.

Studies conducted in Ghana and other LMICs reveal that maternal education, cost of accessing ANC, family size, history of obstetric complications are associated with utilisation of ANC [12–15, 17–20]. However, only few of these studies focus on factors associated with the initiation of ANC using mix methods. This study determined the gestational age of pregnancy at first ANC attendance. It also explored factors that inform initiation of ANC among women who had delivered 6 months prior to data collection. The findings of this study will contribute to literature, inform policy and interventions to encourage women to initiate ANC from the first month of pregnancy for best pregnancy and birth outcomes not only in Ghana but other LMICs and beyond.

## Methods

### Study design and site

This cross-sectional study was conducted in 2016 in the Builsa South District of the Upper East Region (UER) of Ghana. It used mixed methods (quantitative and qualitative) to collect data from women who had delivered within the previous 6 months.

The district is predominantly rural with a population of 38,298 projected from the 2010 Ghana’s Population and Housing Census. Women within reproductive age (15–49 years) form 24% of the total population. The district was selected based on the consistent low (less than 40%) first trimester ANC attendance between 2010 and 2014. Compared to the national average of 64% of pregnant women who started ANC before the fourth month of pregnancy in 2014, Builsa South recorded 37.5% first trimester attendance [15, 16]. The predominant ethnic group is Builsa with few Mamprusis, Sissalas and Fulani nomads in sections of the district. Christians, adherents to traditional religion and Muslims are the dominant religious groups in the district. The population is generally dispersed with poor road network; making access to health care services by some communities difficult especially during the rainy season [16]. The district has 24 primary schools, 15 junior high schools (JHS) and one senior high school (SHS).

Builsa South district is divided into six sub-districts by the GHS for easy management. Concerning health infrastructure, the district has three health centres and 14 CHPS compounds spread across the sub-districts.

### Sampling procedure and selection of survey participants

A single population proportion formula was used to calculate the sample size for quantitative data collection: N = (Z^2^_1 − α/2_) P (1 − P)/ d^2^; because the outcome variable is categorical [23]. Where P is the estimated proportion of women who initiate ANC in the first trimester, d is the acceptable margin of error. The following assumptions were made: proportion of first trimester registrants is 49% based on findings from a previous study in Ghana [17], an acceptable margin of error (d) is 5%, (Z_1 − α/2_) is 1.96 at 95% confidence interval. With a non-response rate of 10%, the final sample size was 431.

A multi-stage sampling technique was used to select women aged 15–49 years old who have delivered in the 6 months preceding the survey. First, a lottery method was used to randomly select one community in each of the six sub-districts. Second, a sampling frame of households with women who had delivered in the past 6 months in the selected communities was developed from the existing filariasis and child health registers in health facilities. Third, systematic random sampling technique was used to select households from the listed households prepared for each community. The eligibility criteria was that the woman should have her ANC record card. Whenever a selected household had two or more eligible respondents, one of them was selected using a lottery method. The number of households selected from each community was proportionate to the number listed on the register.

### Quantitative data collection and analysis

A structured questionnaire, developed for this study [Additional file 1:] was administered by experienced data collectors and supervisors in English or Buili (the local language) based on participants’ preference. The questions were based on published studies [12–14, 18–21, 24–31]. The data collected covered the socio-demographic characteristics and obstetric history of respondents, gestation of pregnancy at first ANC and factors associated with initiation of ANC. The gestation of pregnancy is stated in calendar months of 4 weeks.

The outcome variable: the gestational age of pregnancy at first ANC visit was dichotomised and categorised into ‘early’ for those who initiated ANC in the first trimester (i.e. by 12 weeks of gestation) and ‘late’ for women who made their first ANC attendance in the second and third trimesters (i.e. after 12 weeks and 24 weeks respectively). The explanatory variables were maternal age, marital status, educational attainment, employment status, religion, ethnicity, parity, health insurance status and exposure to information on ANC.

Quantitative data was analysed using STATA version 13. Crosstabs, Chi-square test, Fishers exact test and multiple logistic regression model were used to analyse the quantitative data. Adjusted odds ratio (AOR), 95% confidence interval (CI) and *p*-value< 0.05 show the strength of the statistically significant association between explanatory variables and the outcome variable. A possible correlation between the exposure variables was explored before including them in the model. None were found to be collinear.

### Selection of focus group discussion participants

Thirty-three eligible participants who could not participate in the survey were purposively selected from one rural and one urban community out of the six compiled community register used in the survey for focus-group discussions (FGDs). The participants were selected based on age, education, marital and employment status, religion, insurance status, parity and having their ANC record book.

### Qualitative data collection and analysis

Four FGDs, each consisting of about eight participants (two for young women aged 18–30 years and two for older women 31–44 years) were conducted in Buili and English using a discussion guide. This was to ensure that younger women were comfortable expressing their views freely during discussions since the presence of older women may hinder them from expressing their views freely on the domain of the discussion: factors that influence ANC initiation.

The FGDs were audio recorded and detailed notes taken to capture participants’ views and non-verbal cues. All recordings were transcribed verbatim and translated using the back translation method. The data was analysed using five-steps thematic analysis approach by Braun and Clarke [32] to generate themes from the data.

#### Data Quality Control

The following measures were taken to ensure that the data is reliable. (1) Back translation method was used to translate the questions and interview guides from English to Buili and back to English to ensure that participants understand the questions as intended. (2) Data collectors and supervisors were trained and equipped with interviewing skills. (3) Simulation interviews were conducted by interviewers to ensure they understand the questions as intended and follow all ethical procedures regarding data collection. (4) Data collection tools were pretested in Builsa North District. (5) Study participants were women who delivered in the past 6 months preceding the survey to minimise recall bias. (6) The first ANC visit, and health insurance status were confirmed using participants’ ANC record books. Both authors met to critically review the transcripts and themes to ensure participants’ views were accurately captured.

### Ethical considerations

The Ghana Health Service Ethics Review Committee approved the study. Participants’ consent was obtained after explaining the purpose of the study to them, their right to withdraw from the study at any time. Anonymity, confidentiality and participants’ comfort was ensured.

## Results

### Socio-demographic characteristics of participants

Of the 431 study participants, 39.4% were aged 25–34 years. Their median age was 28 years and range 23–35 years. Also, 58.9% of the participants had some form of formal education, 41.1% have never attended school and 62.2% were Christians. Almost all participants (97.7%) were Builsa, 81.4% were employed (i.e. self-employed and informal sector employees) and 18.6% unemployed (i.e. women not engaged in any activity that gives them income). Also, 24.4% had two living children, 38% more than four and 17.8% had 5–9. Majority (59.6%) had valid health insurance cards during their recent pregnancy (Table 1).

**Table 1:** Socio-demographic characteristics and insurance status of survey participants

Variables	*n* = 431(%)
Age	
Median age = 28 years, range = 23–35 years	
15–24	134 (31.1)
25–34	170 (39.4)
35–44	111 (25.8)
45–49	16 (3.7)
Marital status	
Single	18 (4.2)
Married	409 (94.9)
Cohabiting	4 (0.9)
Religion	
Traditional	118 (27.4)
Christian	268 (62.2)
Moslem	45 (10.4)
Educational level	
No formal education	177 (41.1)
Primary	138 (32.0)
Middle/Junior High School	68 (15.8)
Secondary	44 (10.2)
Tertiary	4 (0.9)
Ethnic group	
Builsa	421 (97.7)
Mamprusi	4 (0.9)
Kassena	4 (0.9)
Sissala	2 (0.5)
Employment	
Unemployed	80 (18.6)
Employed	431 (81.4)
Number of living children	
1	89 (20.7)
2	105 (24.4)
3	73 (16.9)
4	87 (20.2)
5–7	63 (14.6)
8–9	14 (3.2)
Health insurance status	
Currently insured (valid NHIS card)	257 (59.6)
Previously insured (expired NHIS card)	99 (23.0)
Never enrolled	75 (17.4**)**

*NHIS* National Health Insurance Scheme

### Obstetric history and antenatal care utilisation

Of the 431 respondents, 98.8% used ANC at least once during their last pregnancy and 68.3% made four and more visits before delivery. Out of the 426 participants, 42.3% started ANC in the first trimester. Also, 68.3% made four to nine visits before delivery and 59.2% went to CHPS compound (Table 2).

**Table 2:** Participants’ obstetric history and antenatal care use

Variable	N = 431(%)
Used ANC during last pregnancy	n = 431(%)
Yes	426 (98.8)
No	5 (1.2)
Gestation of pregnancy (reported in months) at 1st ANC visit	n = 431(%)
1	38 (8.9)
2	34 (8.0)
3	108 (25.4)
4	193 (45.3)
5	45 (10.6)
6	5 (1.2)
7 and above	3 (0.7)
Gestation of pregnancy at first ANC visit	*n* = 426(%)
First trimester	180 (42.3)
Second trimester	243 (57.1)
Third trimester	3 (0.7)
Source of ANC services for last pregnancy	n = 426(%)
Hospital	1 (0.2)
Health Centre	171 (40.1)
CHPS only	252 (59.2)
CHPS & TBA	1 (0.2)
TBA only	1 (0.2)
Number of ANC visits before delivery	n = 426(%)
1	1 (0.2)
2	14 (3.3)
3	120 (28.2)
4–9	291 (68.3)

*ANC* antenatal care

CHPS community-based health planning and services

*TBAs* traditional birth attendants

### Predictors of early antenatal care attendance

Bivariate analysis of the quantitative data shows a significant relationship between the explanatory variables: religion, educational attainment, and employment status, number of living children, health insurance status and age and timing of initiating ANC while marital status, ethnicity and exposure to ANC information were not. Regarding religion, more Christians (54.9%) compared to 22.2% Muslims and 20.9% traditionalists initiated ANC in the first trimester. Initiating ANC in the first trimester decreases with age; 80% of older women (45–49 years) initiated ANC late compared to 44% of 15–24 year olds (*p* < 0.05). As expected the insured and women who attained formal education initiated ANC early (*p* < 0.05) (Table 3).

**Table 3:** Factors associated with first antenatal care attendance

Exposure variable	Gestation of pregnancy (trimester) at first ANC visit			
	N = 426	Early n(%)	Late n(%)	χ^2^(p < 0.05)
Age	n = 426			
15–24	134	75 (56.0)	59 (44.0)	16.707 (0.001)
25–34	173	65 (37.6)	108 (62.4)	
35–44	104	37 (35.6)	67 (64.4)	
45–49	15	3 (20.0)	12 (80.0)	
Marital status	n = 426			
Single	17	10 (58.8)	7 (41.2)	^a^2.447 (0.338)
Married	405	169 (41.7)	236 (58.3)	
Cohabiting	4	1 (25.0)	3 (75.0)	
Occupation	n = 426			
Unemployed	78	17 (21.8)	61 (78.2)	17.47(< 0.001)
Employed	348	163 (46.8)	183 (53.2)	
Educational status	n = 426	20 (11.6)		
No education	172	62 (44.9)	152 (88.4)	^a^154.183(< 0.001)
Primary	138	53 (77.9)	76 (55.1)	
Middle/Junior High	68	45 (93.8)	15 (22.1)	
Secondary and above	48		3 (6.2)	
Religion	n = 426			
Traditional	115	24 (20.9)	91 (79.1)	46.352(< 0.001)
Christian	266	146 (54.9)	120 (45.1)	
Moslem	45	10 (22.2)	35 (77.8)	
Number of living children	n = 426			
1	89	47 (52.8)	42 (47.2)	26.466(< 0.001)
2	104	54 (51.9)	50 (48.1)	
3	72	34 (47.2)	38 (52.8)	
4	85	30 (35.3)	55 (64.7)	
5–7	62	13 (21.0)	49 (79.0)	
8–9	14	2 (14.3)	12 (85.7)	
Ethnicity	n = 426			
Builsa	416	177 (42.6)	239 (57.4)	^a^3.089 (0.392)
Mamprusi	4	0 (0.0)	4 (100.0)	
Kassena	4	2 (50.0)	2 (50.0)	
Sissala	2	1 (50.0)	1 (50.0)	
Health insurance status	*n* = 429			
Insured	257	130 (50.6)	127 (49.4)	12.180 (0.001)
Not insured	172	51 (29.9)	121 (70.1)	
Exposure to ANC information	426			
Yes	408	171 (41.9)	237 (58.1)	0.462 (0.498)
No	18	9 (50.0)	9 (50.0)	

^a^Fishers Exact test

*ANC* antenatal care

All explanatory variables that are significantly associated with early ANC attendance were put in a multiple logistic regression model while controlling for confounders that also affect ANC use in Ghana. The results suggest that only educational attainment, employment status and number of living children were important predictors of initiating ANC in the first trimester. Women with junior high education had 57.5 higher odds [(AOR = 57.5, CI = 18.50–178.73, *p* ≤ 0.001)] and those who attained secondary education and above had 163.2 higher odds [(AOR = 163.2, CI = 34.94–762.48, p ≤ 0.001)] of initiating ANC early compared to women with no education. Also, women with two, three and four to nine living children were 4.3, 3 and 3.5 times more likely to access ANC in the first trimester compared to first time pregnant women. There was however no difference between first time mothers and women with five or more children regarding early initiation of ANC (*p* > 0.05). Also, employed women have 3.6 higher odds of initiating ANC early compared to the unemployed (Table 4).

**Table 4:** Determinants of early antenatal care attendance

Exposure variable	ANC attendance at first trimester	
	AOR (CI = 95%)	*P*-value
Age		
15–24	1.00	
25–34	0.9 (0.41–2.14)	0.872
35–44	1.2 (0.41–3.31)	0.772
45–49	0.3 (0.04–2.35)	0.245
Occupation		
Unemployed	1.00	
Employed	3.6 (1.50–8.76)	0.004
Educational status		
No education	1.00	
Primary	5.6 (2.82–11.25)	< 0.001
Middle/Junior High	57.5 (18.50–178.73)	< 0.001
Secondary and above	163.2 (34.94–762.48)	< 0.001
Religion		
Traditional	1.00	
Christian	1.6 (0.77–3.16)	0.198
Moslem	0.6 (0.20–1.86)	0.352
Number of iving children		
1	1.00	
2	4.1 (1.35–12.48)	0.013
3	3.0 (1.90–10.04)	0.047
4	3.5 (1.00–12.23)	0.035
5–9	1.1 (0.25–4.46)	0.946
Health insurance status		
Insured	1.00	
Not insured	1.7 (0.82–3.35)	0.135

*ANC* antenatal care

*AOR* adjusted odds ratio

*CI* confidence interval

### Understanding the timing of first antenatal care attendance

This section presents qualitative data from 33 FGD participants. Sixteen, ten, six and one had no education, primary, junior high and secondary education respectively.

Both users and non-users of ANC mentioned a variety of often interlinked factors that inform initiation of ANC. The themes generated from the data were categorised into two: (1) Factors that influence early initiation of ANC. (2) Factors that influence late initiation of ANC.

### Factors that inform early initiation of antenatal care

The factors that influenced early initiation of ANC were categorised into two sub-themes: visible signs of pregnancy and sickness.

#### Visible signs of pregnancy

When asked about the ideal time for initiating ANC, many participants mentioned 3 months and above. They emphasised that timing of the first ANC depends basically on when the visible signs of pregnancy especially protruding stomach occurs. They stated that though they know initiating ANC early is good, they wait for others to notice that they are pregnant before starting ANC. The question many of them asked is: why should I be bothered about ANC if there is no sign of pregnancy? The following were their response to the question what informs their first ANC attendance.

*If the signs of pregnancy especially protruding stomach which occurs usually from the third, fourth or fifth month shows, then you are sure that it is pregnancy and you can start ANC. The important thing is to wait and be sure it is pregnancy then you are confident to start ANC.*

*Initiating ANC in the first three months is important to ensure that the pregnancy is safe. The nurses and doctors will check whether there is something wrong with you and the baby. If the baby is not lying in the correct position they will position it well and correct any problem the mother has. Also, diseases like HIV that you give to the baby is prevented. But I think you don’t have to rush and go; four or five months is okay for initiating ANC. I say this because some people do not show the signs of pregnancy such as vomiting, spitting and the stomach does not protrude early. Such people will have to wait till four, five or even six months when everything is visible.*

#### Sickness

Participants stated that many people initiate ANC early, only when they are sick or experience any pregnancy-related health problems. Early initiators emphasised that they go to the ANC early if the pregnancy is under threat or they are sick in the following comments.

*Pregnancy-related sickness prompt women to go to the ANC on time so that they get medicine to ensure the pregnancy is safe. But if the pregnancy does not worry you there is no motivation to go to the hospital. Others, especially young women do not even realize they are pregnant until it is three or four months.*

*Women initiate ANC early mostly because they are sick; either they are vomiting or something is wrong with the pregnancy. So they see the need to go to the ANC early even in the first and second months when they are facing some health problems.*

### Factors associated with late initiation of antenatal care

Discussions with participants revealed that women in the study district generally appreciate the value of initiating ANC early but do not practice what they know. They wait till the third, fourth and fifth months of pregnancy before accessing ANC. The reasons for initiating ANC late were categorised into five main sub-themes: healthy, so do not value the benefits of initiating ANC early, desire to avoid embarrassment associated with the pregnancy, unplanned pregnancy, cost of accessing ANC and traditional rites and practices.

#### Healthy; do not value the benefits of early antenatal care

The absence of complications was mentioned as the main reason of not seeking care early. According to many late initiators of ANC, when healthy and symptoms of pregnancy are not noticeable, they did not see the need to seek care in the first trimester. These category of participants’ responses suggest that though they know the importance of early ANC, they lack adequate knowledge about its benefits of seeking ANC in the first trimester. Their view is that healthy pregnant women do not need to initiate ANC early; they can wait till the signs of pregnancy begin to show. Their typical comments are as follows:

*Though I noticed that I was pregnant, I was not sick so I felt there was no need to go for ANC. Normally, if you are not sick, nobody; not even your husband will ask you whether you have gone to the hospital. So if you are healthy then you go late around four to five months. This is the time when your family also thinks you should start attending ANC and asks whether you have started ANC.*

*Accessing ANC early is good but it is only necessary for those who have problems with the pregnancy. When you are not sick and go about your daily activities, you think you are strong and can carry the pregnancy so you go to ANC when you feel the baby in the stomach.*

*When you are not sick and you go to ANC early, the nurses normally ask why you think you are pregnant. Then you do not really know what to say. So wh*y *should we be bothered to go for ANC when you don’t have problems with the pregnancy and everything is fine*.

*Also, we [older women] depends on previous experience and think we are fine and will not experience any health problem. So we wait until the pregnancy is obvious to everybody around us before we initiate ANC. Some of us know that we have to go to the hospital early but do not. We only go for ANC close to the time of delivery because we think we are experienced*.

#### Fear of being embarrassed

The participants explained that they initiated ANC late to avoid embarrassment related to the pregnancy. Their fear of being embarrassed was based on two issues. First, not knowing how to answer nurses who will question them why they are at the ANC clinic. Second, many participants perceived pregnancy test as not always reliable so do not want to rely on only the results to initiate ANC. They prefer waiting till the signs of pregnancy are clear. They argued that it is better to wait until you stop having menstrual period for 2 or 3 months and your stomach begin to protrude before letting others know about the pregnancy. They reasoned that you could be embarrassed if pregnancy is confirmed and it later turn out to be false. The typical comments were:

*It is better to go to the ANC late than to be embarrassed by the nurses. When you go early, that is, first or second month or even third month when your stomach is not protruding the nurses will ask you why have you came to the ANC clinic, then you are not sure of what explanation to give and feel embarrassed.*

*You might say you are pregnant and go to the hospital early, they have things (kits) they use to test for pregnancy. Sometimes they can confirm that you are pregnant but later on you will not see any pregnancy. So if you rush to check and break the news to your husband that you are pregnant and later it turns out to be false, he will not understand. You can be summoned to a family meeting to explain where the pregnancy has gone to. They might even accuse you of aborting it. Then you embarrass yourself and it becomes difficult for you to explain.*

*Sometimes there could be miscarriage early in the pregnancy. So, you must be sure you have missed your menses three or four months to be sure you are pregnant before you inform your husband and family members, then you start ANC. Otherwise your husband and others may think you are deceiving them.*

#### Unplanned pregnancy

An unplanned pregnancy often takes away the joy of being pregnant. Such pregnancies are not valued and given the required attention compared with an expected pregnancy. The following comments highlight why pregnant women delay initiating ANC.

*I didn’t go to ANC early because I was not expecting the pregnancy. Generally, if pregnancy comes unexpectedly, both the man and the woman become angry with themselves. It is often considered as carelessness on the part of the woman so I hid it and did not attend ANC early.*

*If you are not happy about a pregnancy, there is nothing to motivate you to go to the hospital. I already have five children. I don’t want people to know that I’m pregnant again. So I kept it to myself to prevent any negative comment from my husband and others. Moreover, I don’t have any complications and I know that I’ll be alright even if I don’t go to the hospital early. So I waited and started ANC when the pregnancy is grown; in the 7th month.*

#### Cost of accessing antenatal care

Though ANC is free under the NHIS, the cost of pregnancy test and other payments, and transportation to the facility is not affordable to all pregnant women. Participants’ comments suggest that despite the free ANC services cost of transport and other payments contribute to starting ANC late.

*Since there is no money, my family thinks the little money they have should be kept for the baby. They expect me to manage the pregnancy and start ANC in the fourth, fifth and even sixth month when the pregnancy is noticed by everybody and sometimes close to delivery. If your husband has no money, they will delay you. They think the money can be saved and used for ANC close to delivery and for delivery expenses.*

*I know ANC is free and I have to start early but I had to pay for the pregnancy test to prove I’m pregnant. This is what delay the start of ANC. I’m not working and didn’t have money to make these expenses. Money for transport was what prevented me from seeking ANC early. I had to wait for my stomach to protrude before asking for money to start ANC.*

#### Traditional rites and practices

The participants mentioned that some traditional rites delay initiation of ANC. These rites are expected to protect the pregnant woman and the foetus from ‘evil eyes’. Many of the participants typically described what usually happens as follows:

*Going to ANC early is okay but there are some traditional rites that are performed before women show that they are pregnant and start ANC. This rite can delay especially for first time pregnant women and thereby delay initiation of ANC visit. They expect you to delay ANC until you go through the rite to protect both the woman and the unborn child from harm.*

*My people belief that pregnancy need to be protected from evil forces. They claim that a pregnant woman must go through certain rites before showing that she is pregnant and going to the health facility. This is done to prevent any ‘evil power’ from causing harm. Older women will frighten you that if you disobey the customs of protecting the pregnancy against harm, the foetus will not grow well. So you become frightened and obey them; and wait till you are taken through the rite before you start ANC. Many of the educated women ignore the rite, but the less educated go through the rite.*

*Sometimes it is the people around you who think you can manage the pregnancy at the early stages and go to the ANC later. In fact many people think a pregnant woman had to wait to be sure she is pregnant before announcing it and then go for the rite before seeking ANC. So if the pregnancy does not worry you, before you realise it’s already four, five or six months*.

## Discussion

The results of this study show that almost all participants (98.8%) use ANC at least once before delivery. However, less than half (42.3%) initiate ANC in the first trimester as recommended by WHO [4–6]. The majority (57.1%) of the participants initiated ANC in the second trimester. These findings support results of previous studies that the number of pregnant women who initiate ANC in the first trimester in many sub-Sahara African countries is low [12–16, 19, 24–31]. A facility data in the studied district in Ghana’s UER shows that less than 40% of the women initiate ANC in the first trimester between 2010 and 2014 [16]. Also, a facility-based study in Northern West Ethiopia found that 47.3% of pregnant women initiated ANC early in 2013 [13].

Drawing on political ecology of health which emphasises that researchers should consider interactions between human beings and their environment to gain insight into structural causality rather than focusing solely on individual self-interested behaviour [33], a number of factors that influence initiation of ANC in a rural district in Ghana are discussed. Our discussion illuminates the effect of context on uptake of international public health policies. It reveals how local level drivers of ANC initiation shape acceptance of global ANC policy within the context of Ghana’s UER. The FGD data shows that individual and contextual factors influence women to either initiate ANC early or late.

Initiating ANC is often prompted by visible signs of pregnancy and sickness. These results corroborate findings from previous studies [20, 21, 29]. Haddrill and others’ qualitative study in South Yorkshire reported that the absence of signs and symptoms of pregnancy delayed ANC initiation [20]. A multi-country study covering Ghana, Kenya and Malawi revealed that health problems whether pregnancy-related or not prompted women to seek ANC early [29]. Another study in Holeta town in Ethiopia also found that most of the ANC non-attendees are in a state of good health [21].

Women’s education the number of living children and being employed have been consistently associated with use of maternal health services. Our results show that the odds of starting ANC in the first trimester was highest among women who have attained secondary and tertiary education. The participants’ responses suggest that they lack adequate knowledge about its benefits of initiating ANC in the first trimester. Their view is that healthy pregnant women do not need to initiate ANC early; they can wait till the signs of pregnancy begin to show. This phenomenon could be due to the fact that educated women are more likely to be exposed to maternal health information that enhances their knowledge on the benefits of initiating ANC early; hence value ANC attendance in the first trimester to achieve best pregnancy and birth outcomes and not wait for visible signs of pregnancy or experience any complication. This finding supports the results of previous studies in Ghana and other parts of Africa that the use of maternal health services increases with attainment of formal education [13, 15, 17, 21, 25, 34, 35]. Nketiah-Amposah and others’ study found a positive relationship between women’s level of education and ANC use [25]. Birmeta et al.’s study in Central Ethiopia also found that reasons for non-attendance of ANC includes absence of illness, no or little knowledge about ANC [21].

Regarding the number of living children, women with two, three or four children were 4.1, 3, 3.5 times respectively more likely to initiate ANC in the first trimester compared to primigravidae women. This result supports a Southern Ethiopian study results that previous ANC use was a positive predictor of initiating ANC early [28].

Late initiation of ANC is attributed to three main factors: individual factors, indirect cost of accessing ANC and traditional rites and practices. Regarding individual factors, five groups of late initiators of ANC could be identified. The first group of late ANC initiators are women who do not have adequate knowledge about the benefits of initiating ANC early. Ndidi and Oseremen study in Nigeria’s Niger Delta also found that late ANC attendance could be due to ignorance or misconceptions of the purpose of, and right time to commence ANC [12]. Banda et al. also found in their study in Zambia that women with adequate knowledge about ANC were likely to initiate care early when pregnant [34]. These results can be described as a case of lacking the correct knowledge that all pregnant women whether healthy or not should initiate ANC in the first trimester to achieve best pregnancy and birth outcomes. The second group are older pregnant women who do not experience any complications in their previous pregnancies. Being familiar with ANC procedures from previous pregnancies and confident that they can manage the pregnancy without experiencing any negative consequences, influence multiparous women with five or more children to seek care late. This result suggests that exposure to ANC information is not enough to influence women to initiate ANC early rather their emotional preparedness and confidence that they can go through the initial stages of pregnancy without seeking care from health professionals and go to the facility late sometimes close to delivery. The third category of late ANC initiators are primigravidae women who are often young. Our quantitative results show that many primigravidae women were less likely to initiate ANC in the first trimester. They disclosed to their partners and other relatives when the signs of pregnancy was obvious. Our results and findings of previous studies indicate that the late disclosure could be either because primigravidae women are inexperienced and might not be familiar with subtle signs of pregnancy to be certain that missing their last menstrual period means they are pregnant so they wait for 3 to 4 months to confirm before disclosing pregnancy in order to avoid embarrassment. Thus, social ramification of pregnancy could lead to hiding it and initiating ANC late especially among adolescents. [28, 29]. A Southern Ethiopian study also found that no experience of previous use of ANC is a positive predictor for seeking care late [28]. The fourth group of late ANC initiators were those who perceive pregnancy test as not always reliable so want to avoid not only embarrassment from health workers but also misunderstanding between them, their spouse and other family members. Thus, mistrust in the accuracy of pregnancy test and avoidance of embarrassment discourage early disclosure of pregnancy and initiation of ANC. The last group of late ANC initiators were those whose pregnancy is unintended. It is the case that desired pregnancies are more cared for by women and their partners, hence initiate ANC early. But if they are unhappy about the pregnancy they delay seeking ANC as long as they are not experiencing any complication. This finding agrees with the findings from previous studies [20, 26, 27]. A Tanzanian study shows that early ANC initiation was highest for intended pregnancy. They observed that women who had unintended pregnancies may not have partner support to seek care early [27]. Haddrill and colleagues’ study in South Yorkshire in the United Kingdom also found that unplanned pregnancy delayed ANC initiation [20]. These results suggest that if pregnancy is not anticipated, social consequences of unwanted pregnancy and emotional demands of motherhood delay ANC initiation.

Second, our qualitative results reveal that though ANC is free under the NHIS policy, cost still remains a barrier in seeking care early. The NHIS covers only the direct cost of ANC, so poor pregnant women though insured, could not afford the indirect cost which includes the cost of pregnancy test, other payment and transport fares. Our quantitative results also show that compared to the unemployed, employed pregnant women have 3.6 higher odds of initiating ANC early. This finding agrees with other studies in sub-Saharan Africa that there is an association between wealth and use of health services [17, 21, 30, 31, 36]. Arthur found in his analysis of the 2008 GDHS that even after free ANC services wealth still has a significant influence on adequate use [30]. A Ugandan study also found that 27.5% of their participants started ANC late because they did not have money for transport to the hospital [31]. Thus, it is worrying that despite the free access to ANC, insured poor pregnant women, often unemployed, are not likely to afford the indirect cost so unable to access care early.

Finally, traditional rites and practices contribute to delay in initiating ANC. Studies have documented that traditional practices hinder seeking modern healthcare promptly. Our results thus support the impact of such practices which still exist especially in rural communities and among adherents of traditional rites in Ghana. This finding supports a rural Cambodian study result that traditional ceremonies most women go through could be a barrier to utilising maternal health services [36]. Banda et al. found that cultural belief and value system of the society in which individuals live influence ANC attendance [35]. These practices undermine efforts to enable all pregnant women initiate ANC early and complete all the necessary obstetric interventions by 21 weeks to prevent adverse pregnancy and birth outcomes [4–6, 9–11].

These contextual factors explain why the removal of financial and geographical barriers through free ANC services under the NHIS and establishment of CHPS compound nation-wide could not ensure that all women attend ANC in the first trimester. Critical analysis of the current results and previous findings suggest that no or low female education is the most important factor that underlies pregnant women’s inability to access ANC early. Educated women seem to have adequate knowledge about the benefits of early ANC attendance, are more likely to afford the indirect cost of accessing ANC without necessarily relying on their spouse for money, ignore the perceived unreliability of pregnancy test results and not yield to the pressure of going through traditional rites that are detrimental to their health. Thus, formal education increases one’s ability to apply health information and make reasonable decisions to attend ANC early to improve pregnancy and birth outcomes.

### Limitation of the study

Two main limitations of this study were identified. First, the study was conducted in only one district, hence the results might not be generalised to the whole country and other LMICs. Second, the sample is made up of only women who attended postnatal clinics so the results might not apply to other categories of post-partum women. The conclusions should therefore be interpreted with caution.

## Conclusion

Initiation of ANC is based on visible signs of pregnancy, particularly protruding stomach and sickness. Education, the number of living children and earning income were the factors significantly associated with initiating ANC in the first trimester. Many are yet to appreciate the need for all pregnant women, even when healthy to initiate ANC in the first trimester. They lack the correct knowledge of the benefits of early ANC attendance. Unplanned pregnancy, embarrassment associated with the pregnancy, indirect cost of accessing ANC in Ghana, traditional rites and practices contribute to late ANC attendance. The findings explain why adverse pregnancy outcomes is still high and calls for adequate investment in policy and health promotion programmes to improve initiation of ANC in the first semester. The intervention programme should include: (1) Health facilities should sensitise key stakeholders including husbands, in-laws, traditional and religious leaders on the benefits of initiating ANC early, dangers associated with late attendance and engage them to address contextual issues that are at odds with early ANC attendance. (2) The GHS should sensitise their staff not to ask questions that will embarrass women who seek ANC in the first trimester. (3) The government of Ghana should ensure that girls are not enrolled in school but kept to complete at least junior high education to better appreciate the benefits of accessing ANC early. (4) Financial barriers to accessing ANC services should be completely removed by expanding NHIS’ benefits to cover the pregnancy test required before enrolment and other payments. Finally, more qualitative studies are needed to assess both community members and health professionals’ acceptability of initiating ANC in the first trimester and explore further traditional rites that undermine efforts towards achieving universal use of ANC in the first trimester.

## Additional file

Additional file 1: Questionnaire administered to women 15–49 years who have delivered 6 months preceding the data collection. The file is a the questionnaire used to collect data on the socio-demographic profile of respondents, use of and timing of initiation of antenatal care. (DOCX 33 kb)

## Data Availability

The data generated and analysed can be requested from the corresponding author.

## Abbreviations

ANC: Antenatal care
AOR: Adjusted odds ratio
CHPS: Community-based health planning and services
CI: Confidence interval
FGDs: Focus group discussions
GDHS: Ghana Demographic and Health Survey
LIMCs: Low and middle income countries
NHIS: National Health Insurance Scheme
P: *P*-value
SBA: Skilled birth attendant
SHISs: Social health insurance schemes
STIs: Sexually transmitted infections
TBAs: Traditional birth attendants
UER: Upper East Region
WHO: World Health Organization

## Footnotes

1 Community-based health planning and services (CHPS) zone is a national programme of community-based care provided by resident nurses referred to as community health officers. CHPS, introduced in 1999, reduces geographical barriers to healthcare access and provides basic level preventive and curative services for minor ailments at the community and household levels.
