# Supplementary material for: “No visible signs of pregnancy, no sickness, no antenatal care”: Initiation of antenatal care in a rural district in Northern Ghana
Source: BMC Public Health. 2019 Aug 13;19:1094. doi: 10.1186/s12889-019-7400-2 (PMC6693094; doi:10.1186/s12889-019-7400-2)
Supplement: Supplementary file 1 — Questionnaire administered to women 15–49 years who have delivered 6 months preceding the data collection. The file is a the questionnaire used to collect data on the socio-demographic profile of respondents, use of and timing of initiation of antenatal care. (DOCX 33 kb) [file 12889_2019_7400_MOESM1_ESM.docx]

## Questionnaire on the use of and timing of initiation of antenatal care administered to women aged 15-49 years

Compound name:…………………………….Community name………………………...

Date of interview……/……/… Interview start and end time……………….

| No | Question | | Code |
| --- | --- | --- | --- |
| SOCIO-DEMOGRAPHIC DATA | | | |
| 1 | Age of respondent at time of delivery (in completed years)………… (confirm from any valid ID) | | Q1age |
| 2 | Marital status  Single………………………………………………………..………………….1  Married……………………………………………………..…..........................2  Cohabiting……… ……………………………………….…………………...3  (CIRCLE ONLY ONE) | | Q2mar_st |
| 3 | Respondent’s occupation  Unemployed…………..……………………………………….………………1  Farmer………………………………………………………………………....2  Trader/Business………………………………………………………………..3  Government employee………………………………………………………...4  Private sector employee……………………………………………………….5  Others (*specify)..*…………………………………………………  (CIRCLE ONLY ONE) | | Q3occup |
| 4 | Highest educational level attained  No education……………………………………………………………….….1  Primary ………………………………………………………………………..2  Middle/Junior High School……………………………………………………3  Secondary……………..………………………………………………………4  Tertiary ………………………………………………………………………..5  (CIRCLE ONLY ONE) | | Q4educ |
| 5 | Religion  Traditional……………………………………………………………………..1  Christian……………………………………………………………………….2  Moslem………………………………………………………………………...3  Others *(specify)..*………………………………………………….  (CIRCLE ONLY ONE) | | Q5relig |
| 6 | Parity (number of living born children the woman has)  1………………………………………………………………………………...1  2…………………………………………………………….…………………..2  3………………………………………………………………………………...3  4………..………………………………………………………………………4  5-7……………………………………………………………...........................5  8-9……………………………………………………………………………...6  10+……………………………………………………………………………..7  (CIRCLE ONLY ONE) | | Q6parity |
| 7 | Ethnicity  Builsa…………………………………………………………………………...1  Kantoosi………………………………………………………………………..2  Mamprusi……………………………………………………….........................3  Kassena ………………………………………………………………………..4  Sissala…………………………………………………………………………..5  Others (*Specify)…………………*………………………….………  (CIRCLE ONLY ONE) | | Q7ethnic |
| 8 | Were you registered unto the National Health Insurance Scheme in your last pregnancy? *(If yes ask to see card)*  Yes…………………………………………………………………….………1  No……………………………………………………………. ……………….2  (CIRCLE ONLY ONE) | | Q8insura  SKIP TO Q10 |
| 9 | Health insurance card validity status ( as at last pregnancy)  Not valid health insurance………………………………………………….….1  Valid health insurance…………………………………………........................2  Not applicable…………………………………………………………………88  (CIRCLE ONLY ONE) | | Q9valdins |
| ATTENDANCE OF ANC AND USE OF ANC | | | |
| 10 | Did you attend ANC for your most recent child [name]?  Yes…………………………………………………………………………….1  No……………………………………………………………..........................2  (CIRCLE ONLY ONE) | Q10anc_at  SKIP TO Q16 | |
| 11 | At what age (in months) of pregnancy did you start ANC for your recent pregnancy?(*comfirm from ANC record book )*  1month……….…………………………………………………………………1  2 months…..……………………………………………………………………2  3 months………………………………………………………………………..3  4 months………………………………………………………………………..4  5 months………………………………………………………………………...5  6 months………………………………………………………………………...6  7 months and above……………………………………………………………..7  Not applicable………………………………………………………………….88  (CIRCLE ONLY ONE) | Q11gest | |
|  |  |  | |
| 12 | Where did you receive ANC services for [name of recent child]? (probe) (Confirm from ANC book)  Health centre…………………………………………………………………….1  CHPS……………………………………………………………………………2  TBA……………………………………………………………………………..3  Hospital…………………………………………………………………………4  Others *(specify)………………………………………………………*………………..  Not applicable…………………………………………………………………..88  (CIRCLE ALL THAT APPLY) | Q12wheranc | |
| 13 | How many visits (excluding visits for own medical care) did you make before delivering [name]?(*confirm with ANC book)*  1……………………………………………………………………………….1  2………………..……………………………………………………………2  3……………………………………………………………………………..3  4……………………………………………………………………………..4  5 and more…………………………………………………………………..5  Not applicable………………………………………………………………88  (CIRCLE ONLY ONE) | Q13visits | |
| 14 | Where you exposed to information on ANC services during your recent  Yes…………………………………………………………………………….1  No…………………………………………………………….......................... 2 | SKIP TO Q13 | |
| 15 | In your opinion at what age of pregnancy should a woman start ANC?  1month……….……………………………………………………………..… ..1  2 months…..…………………………………………………........................... .2  3 months…………………………………………………………………………3  4 months…………………………………………………………………………4  5 months…………………………………………………………………………5  6 months…………………………………………………………………………6  7 months ………………………………………………………………………...7  8 months and above……………………………………………………………..8  I don’t know…………………………………………………………………….9  (CIRCLE ONLY ONE) | Q17oppanc | |
| 16 | Is there any question (s) or contribution you will want to make towards this study? |  | |
